# Translational symmetry in convolutions with localized kernels causes an implicit bias toward high frequency adversarial examples

**DOI:** 10.3389/fncom.2024.1387077

**Published:** 2024-06-20

**Authors:** Josue O. Caro, Yilong Ju, Ryan Pyle, Sourav Dey, Wieland Brendel, Fabio Anselmi, Ankit B. Patel

**Affiliations:** ^1^Department of Neuroscience, Baylor College of Medicine, Houston, TX, United States; ^2^Department of Electrical and Computer Engineering, Rice University, Houston, TX, United States; ^3^Manifold AI, San Francisco, CA, United States; ^4^Max Planck Institute for Intelligent Systems, University of Tübingen, Tübingen, Germany; ^5^Department of Mathematics, Informatics and Geosciences, University of Trieste, Trieste, Italy; ^6^Massachusetts Institute of Technology (MIT), Cambridge, MA, United States

**Keywords:** adversarial examples, implicit regularization, neural networks, convolutional architectures, Uncertainty Principle

## Abstract

Adversarial attacks are still a significant challenge for neural networks. Recent efforts have shown that adversarial perturbations typically contain high-frequency features, but the root cause of this phenomenon remains unknown. Inspired by theoretical work on linear convolutional models, we hypothesize that *translational symmetry in convolutional operations* together with *localized kernels implicitly bias the learning of high-frequency features*, and that this is one of the main causes of *high frequency adversarial examples*. To test this hypothesis, we analyzed the impact of different choices of linear and *non-linear* architectures on the implicit bias of the learned features and adversarial perturbations, in spatial and frequency domains. We find that, independently of the training dataset, convolutional operations have higher frequency adversarial attacks compared to other architectural parameterizations, and that this phenomenon is exacerbated with stronger locality of the kernel (kernel size) end depth of the model. The explanation for the kernel size dependence involves the Fourier Uncertainty Principle: a spatially-limited filter (local kernel in the space domain) cannot also be frequency-limited (local in the frequency domain). Using larger convolution kernel sizes or avoiding convolutions (e.g., by using Vision Transformers or MLP-style architectures) significantly reduces this high-frequency bias. Looking forward, our work strongly suggests that understanding and controlling the implicit bias of architectures will be essential for achieving adversarial robustness.

## 1 Introduction

Despite the enormous progress in training neural networks to solve hard tasks, they remain surprisingly and stubbornly sensitive to imperceptibly small perturbations known as *adversarial examples*. Extensive research has been conducted on the nature and structure of adversarial examples, as evidenced by studies such as Goodfellow et al. (2014), Tanay and Griffin (2016), Bubeck et al. (2018), Fawzi et al. (2018), Gilmer et al. (2018), Schmidt et al. (2018), Ford et al. (2019), Ilyas et al. (2019), and Mahloujifar et al. (2019). One notable finding from experiments is that adversarial examples often exhibit a significant amount of high-frequency energy content (Yin et al., 2019) but their precise origin and nature remain obscure. In this context, a natural questions emerges: does this phenomenon depend on the neural network architecture or on the training dataset?

### 1.1 Influence of the dataset on the nature of the adversarial examples

Previous studies have demonstrated that adversarial examples are not random perturbations of the input space; rather, they contain dataset-specific information that reveals class decision boundaries (Ilyas et al., 2019). This raises the question: “*Do high-frequency energy concentration in adversarial examples reflect specific task- and data-dependent learned features?*”. Interestingly, Wang et al. (2020) showed that high-frequency features are crucial for achieving high generalization performance in various models trained on CIFAR10. They argue that learning high-frequency features is a data-dependent phenomenon, as models relying on lower-frequency features exhibited lower accuracy. Previous research has also demonstrated that the sensitivity to certain frequency-based features can be modified by reducing their reliability through data augmentations in the dataset (Geirhos et al., 2018; Hermann et al., 2020; Li et al., 2022). Maiya et al. (2021) provided evidence that different datasets produce adversarial examples with varying concentrations of energy in the frequency domain that correlate with the dataset statistics.

*Taken together, these findings suggest that the selection of features, particularly high-frequency features, is largely influenced by dataset statistics and that this bias, in turn, affects the nature of adversarial examples*.

### 1.2 Influence of the neural network implicit bias on the nature of the adversarial examples and the Implicit Fourier Regularization hypothesis

In many cases, datasets contain multiple features that are correlated with the target function and the learned weights to detect those features. A natural question is therefore: “*Why does a particular model tend to use frequency-based features, particularly high-frequency features, and how is this related to the nature of adversarial attacks?*”. Various theories have been proposed to explain the robustness and generalization of neural networks from a frequency perspective. One example is Universal Adversarial Perturbations, a method used to determine the directions in input space that neural networks are sensitive to Tsuzuku and Sato (2019). The authors' findings highlight the importance of the model choice for robustness, as they discovered that convolutional neural networks exhibit sensitivity to noise in the Fourier Basis, unlike other models such as MLPs.

In addressing the aforementioned question, we adopt a similar, but more general, approach that relies on the concept of “implicit bias.” Implicit bias in machine learning refers to the phenomenon where the training process of an overparameterized network, influenced by factors including the choice of model architecture and parametrization (Gunasekar et al., 2018; Yun et al., 2020), the initialization scheme (Sahs et al., 2020a), and the optimization algorithm (Williams et al., 2019; Sahs et al., 2020b; Woodworth et al., 2020), naturally favors certain solutions or patterns over others, even in the absence of explicit bias in the training data. The implicit bias of state-of-the-art models has been shown to play a critical role in the generalization of deep neural networks (Arora et al., 2019; Li et al., 2019). Recent theoretical work (Gunasekar et al., 2018) on *L*-layer *deep linear networks* proved that (i) fully connected layers induce a depth-independent ridge (ℓ_2_) regularizer in the spatial domain of the network weights whereas, surprisingly, *full-kernel convolutional layers* (i.e., where the support of the kernel weights is the full image, in contrast to local kernels) induce a depth-dependent *sparsity* (ℓ_2/*L*_) regularizer in the weights *frequency* domain. The hypothesis we aim to test is that the learned weights, which determine the features detected in the dataset to solve the task, also influence the characteristics of adversarial examples.

At this point, it is important to note that linear convolutional models differ from the high-performance convolutional neural networks (CNNs) typically used in practical applications. Nevertheless, we postulate that similar mechanisms of implicit regularization might be operating in deep nonlinear models with local convolutions. In particular, we suggest that the high-frequency nature of adversarial perturbations arises not solely from the dataset statistics but also from the implicit bias induced by the specific architectural choice. To formalize this hypothesis, we introduced the Implicit Fourier Regularization (IFR) hypothesis:

*Translational symmetry in convolutional operations together with localization of kernels introduces an implicit regularization in the frequency content of the network weights and adversarial attacks, leading to a preference for higher frequencies*.

The IFR hypothesis suggests that in datasets where high-frequency features are important for the task, models using convolutional parametrization with local kernels tend to have a bias toward learning these features. As a result, adversarial perturbations generated by these models also tend to exhibit high-frequency components. More broadly, our research establishes a connection between the implicit regularization arising from model parametrization and the structure of adversarial perturbations.

## 2 Methods

### 2.1 Neural network training

Each architecture was defined by the number of hidden layers, and non-linearities (see Supplementary Table 1). In terms of hyperparameters, we tuned the maximum learning rates for each model by starting from a base learning rate of 0.1, and then, if there were visible failures during training (most commonly, the model converging to chance performance), we adjusted the learning rate up/down by a factor of 10 or 50. Amongst the model architectures we explored, the only hyper-parameter that was tuned was the learning rate. The final values of the learning rates after search are detailed in Supplementary Table 5. In addition, all the models were trained with linearly decaying learning rate follow 0.3 factor for each epoch and resetting the learning rate back to max when the model was trained at least 20 epochs. All the models were trained on a single GTX 1080 Ti for at least 40 epochs (30–120 GPU minutes), and we choose the epoch with the highest validation set accuracy for further experiments (see hyperparameters of training and accuracy on Supplementary Section 1.3).

### 2.2 Adversarial attack generation

We used the Foolbox package (Rauber et al., 2017; MIT license) to generate adversarial perturbations δ for every example in the test set for a fully trained model (PGD-Linf, PGD-L2, PGD-L1, Kurakin et al., 2016, BB-Linf, and BB-L2, Brendel et al., 2019). Finally, we computed the 2-D Discrete Fourier spectrum $\hat{\delta }$ :=Fδ of the perturbation δ. Details of the attacks are available in Supplementary Table 10.

### 2.3 Model predictor calculation and Toeplitz matrix

In our work we started by considering linear networks ϕ:ℝ^*d*^ → ℝ given by


(1) $$\phi (x)=\beta_{L}^{T}x:\text{​​}=(\prod_{l=1}^{L}W_{l})x$$

where *x* is a vector in ℝ, $W_{l}\in {\mathbb{R}}^{d\times d}$ represents the network's weights and *L* the number of layers. β represents the model predictor and contains the information about the type of features the network learn to detect in the input. The characterization of the learned β will be therefore one of the focuses of this work as it determines the way the model is extracting information from the input and also its biases. For its computation we used two different methods. For the linear models, we transformed the weights of every architecture into their matrix form. For example for the convolutional operation, we generated a Toeplitz matrix per convolutional filter and then calculated the dot product of the first *l* matrices to get the β_*l*_ [or for all *l* = 1, ⋯ , *L* to get the input-output function β, see Equation (1) and Gunasekar et al., 2018]. For the nonlinear models, because the nonlinearities do not allow us to use the weights directly, we decided to use a proxy, the saliency map. The saliency map is the gradient ($\frac{d\phi }{dx}$) of the function [ϕ(*x*)] with respect to the input image (*x*). In the linear case, these gradients are exactly the weights of the function β (up to a constant), which we confirmed using the Toeplitz computation above. For the nonlinear models, because the weights used changed per example, the gradient gave us a good approximation of those weights.

### 2.4 Generation of hidden shortcut features

**Figure 1:**
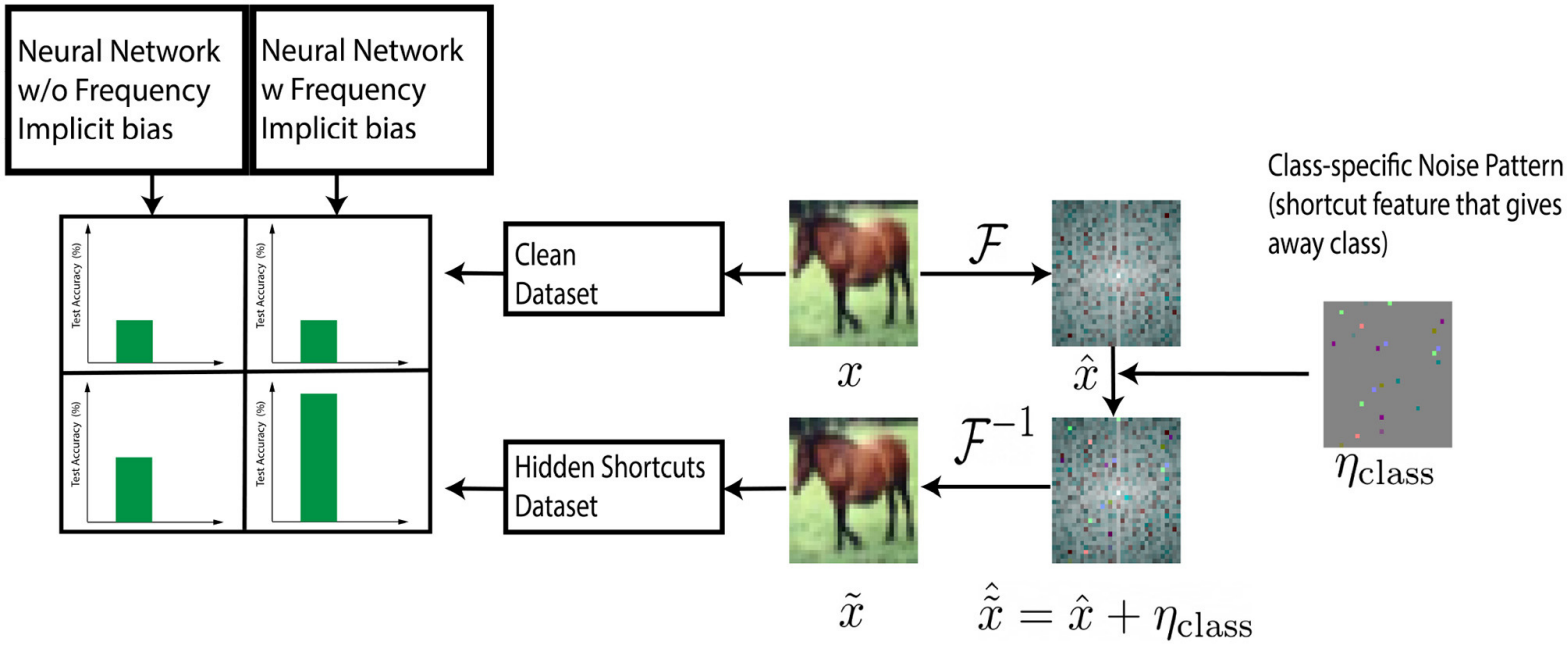
Steganography experiment. Class specific information η_*class*_ is introduced into Fourier Spectrum of training set image ($\hat{\tilde{x}}=\hat{x}+\eta_{\text{class}}$). Models with sparse frequency regularizer (full and local kernel convolutional models) take more advantage of hidden shortcut features in the dataset leading to higher test accuracy.

Our technique draws inspiration from the field of *steganography*, which introduces visually imperceptible features in images (Cheddad et al., 2010). Here we describe how we added class-correlated features in the Fourier space of the train and test set images to highlight biases of the model representation in Fourier space see also Figure 1. Those features are generated in the form of a noisy matrix (added to the image) with specific frequency characteristics (High, Medium, and Low). Below we detail how we generated those matrices.

For each class, we sampled a 3 × 32 × 32 matrix of scalars (image dimension) from a Gaussian distribution with mean of 0 and standard deviation of 1, one per class (*N*_*class*_). Then we scaled the features by a scalar factor ϵ. Next, we generated masking matrices (*M*_*class*_) of the same size. Subsequently, we filtered the *N*_*class*_ matrices by computing the Hadamard product of them with *M*_*class*_ masking matrices for low, medium and high frequencies. Finally, these class-specific features were added into the Fourier spectrum of CIFAR-10 train and test images corresponding to their respective classes. The mathematical definitions are as follows:


$$\begin{array}{l} N_{\text{class}}\sim N(0,1)\\ M_{class}=\text{Frequency}M_{\text{class}}=\{\begin{array}{ll} \text{Inside frequency range} & 1\\ \text{Outside frequency range} & 0 \end{array}\\ \eta_{\text{class}}=M_{\text{class}}⊙(\epsilon *N_{\text{class}}),\;\hat{\tilde{x}}=\hat{x}+\eta_{\text{class}} \end{array}$$

## 3 Experimental results

Next, we will test different implications of the IFR principle. Specifically:

- In Section 3.1 we analyze the implicit bias in the learned weights of a trained network (extending the validity of the results in Gunasekar et al., 2018 to the non-linear case) and its relation with the adversarial perturbations. We do so for a range of architectures (convolutional or fully connected, deep or shallow, linear or not linear) where the *support of the weights is the full image (fully connected or full kernel models)*.
- In Section 3.2 we directly test the IFR hypothesis and focus on convolutional architectures with *local kernels*. Specifically, we analyze the influence of different levels of locality of the convolutional kernel on the Fourier spectrum of the network learned weights and of the associated adversarial attacks. To gain more insight on the nature of the bias we also perform the same analysis focusing on importance of *convolutional translational symmetry* due to weights sharing. Finally, for linear models, we propose a theoretical explanation of the experimental results based on the uncertainty principle.
- In Section 3.3 we further test the models' Fourier spectral bias in non-linear models, injecting *frequency-targeted shortcuts* in the dataset and analyze to which extent different models take advantage of such features.
- In Section 3.4, we evaluate if the results obtained in the previous sections extend to a range of complex models trained on Imagenet. Moreover we consider other state of the art architectures that are not-convolutional, such as transformers, and compare their frequency bias with that of convolutional models.

### 3.1 Full models analysis: relation between implicit bias and adversarial perturbations

To establish if there exists a relationship between the network's implicit regularization and the adversarial perturbations, we started, as previously mentioned, from the recent theoretical results in Gunasekar et al. (2018) where the authors considered the linear network as Equation (1). They prove that:

- when no restrictions is imposed on the *W*_*l*_ matrices (fully connected layers), training with stochastic gradient descent naturally converges to a solution with minimal ||β_*L*_||_2_ norm.
- when the *W*_*l*_ matrices are convolutional (with kernels support the full image, full kernel) the training converges to a solution with minimal $||{\hat{\beta }}_{L}|{|}_{\frac{2}{L}}$ norm where ${\hat{\beta }}_{L}$ is the Discrete Fourier Transform of β_*L*_.

In other words, changing the parametrization of the linear layer of the model in Equation (1) induces different learned features β.

Here, through experiments, we: (1) confirm these findings; (2) extend them to non-linear models; (3) test that a similar regularization is also present in the adversarial perturbations δ generated from each considered model. We started this analysis considering the two linear architectures discussed in Gunasekar et al. (2018), namely fully connected and full kernel convolutional models. Subsequently, we considered non-linear architectures including shallow versions (with one hidden layer) and deep versions (with three hidden layers) of these models. Moreover, we trained the models on five different datasets: CIFAR-10 (Krizhevsky and Hinton, 2009), CIFAR100 (Krizhevsky and Hinton, 2009), MNIST (LeCun and Cortes, 2010), FashionMNIST (Xiao et al., 2017), and SVHN (Netzer et al., 2011) using PyTorch (Paszke et al., 2019). Throughout the paper, we employed the PGD attack, which is considered a standard attack in the field (see Section 2 for details).

**Figure 2:**
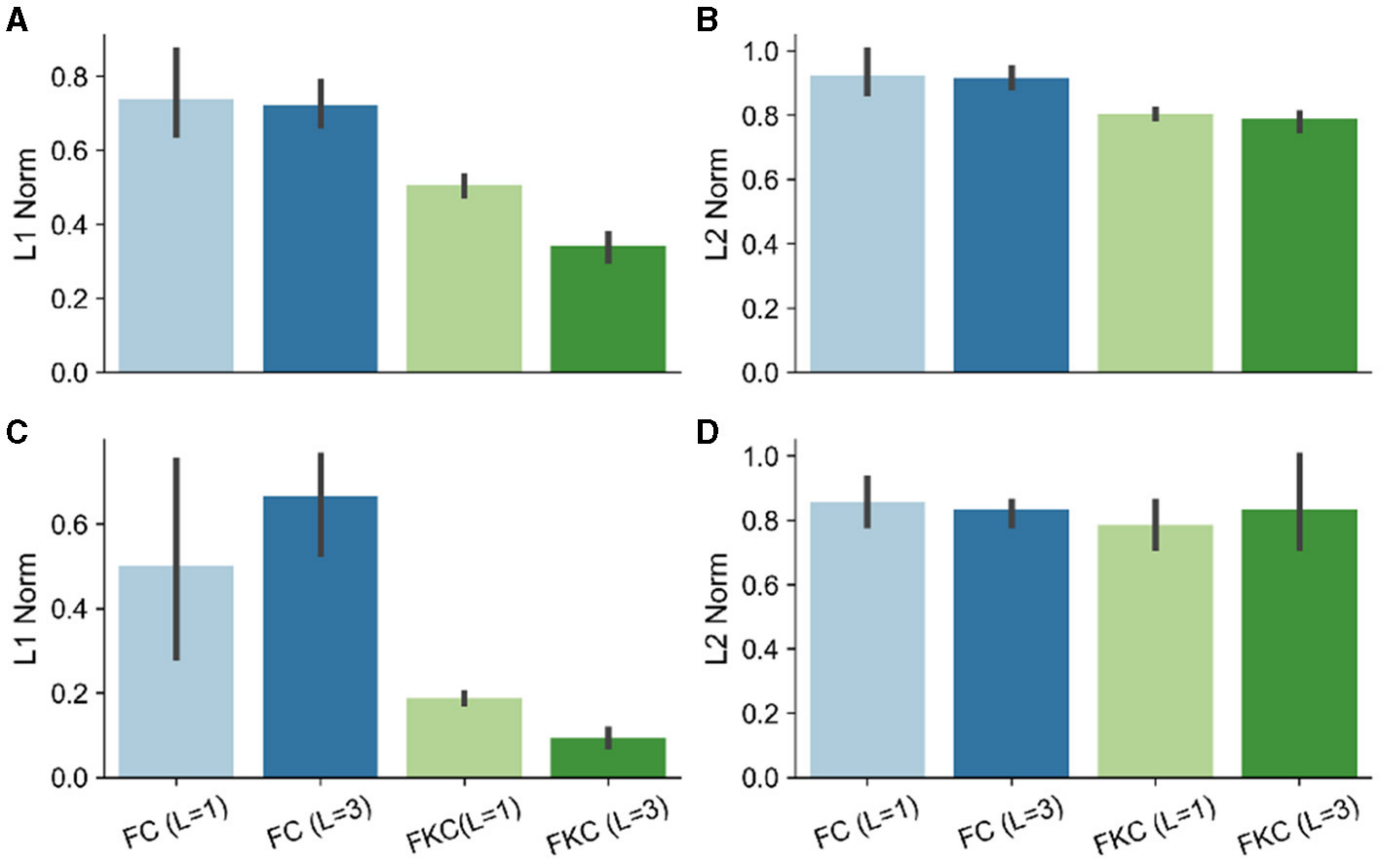
**(A)** ℓ_1_ norm of $\hat{\beta }$; **(B)** ℓ_2_ norm of $\hat{\beta }$; **(C)** ℓ_1_ norm of the averaged $\hat{\delta }$ perturbations; **(D)** ℓ_2_ norm of the averaged $\hat{\delta }$ perturbations. FC, Fully Connected; FKC, Full Kernel Convolutional; L, number of layers.

In Figure 2 we report the values of the (max-normalized) ℓ_2_ (of β, δ) and ℓ_1_ (of $\hat{\beta },\hat{\delta }$) norms for the different considered architectures. We observe that :

- The results in Gunasekar et al. (2018) are confirmed and extended to the non-linear case: 1) the ℓ_1_ norm of $\hat{\beta }$ in the case convolutional full kernel architectures is depth dependent (a); 2) fully connected networks have ℓ_2_ norms of β that do not change with depth (b).
- The same pattern holds for the average across adversarial perturbations associated to each model choice (c,d).

### 3.2 Translational symmetric convolutional models with localized kernels: testing the IFR hypothesis

One limitation of the analysis done in Gunasekar et al. (2018), and in the previous section, is that full kernel convolutional layers are not often used in common state-of-the-art architectures. Non-linear convolutional models with localized kernels are usually employed instead. Moreover the theory in Gunasekar et al. (2018) only specifies a bias toward $l_{\frac{2}{L}}$-sparsity in the frequency domain of the weights for linear convolutional networks with no information about the distribution of those frequencies. In this section we fill this gap and focus on convolutional non linear models with local kernels and analyze and compare the *energy distribution in the frequency domain* of the network's weights β and the adversarial perturbations δ. Moreover, to investigate if *convolutional weights sharing*, i.e., translational symmetry, is playing a major role in determining such energy distribution, we consider a third version of the model with local kernels but no convolutional sharing of the weights, which we call *locally connected* model.

The results, reported below, are directly related to the main claim of the paper, the IFR hypothesis, i.e., that convolutional operations with decreasing kernel size favor higher frequencies learning in the network weights and adversarial attacks. In specific, we consider convolutional non linear models (of different depths and non-linearities) with (1) local (fixed size) or (2) full kernels and (3) locally connected models. For the associated β and δ we then:

- Calculate the half power frequency (*f*_50_), i.e., the frequency at which we accumulate the 50% total energy and average across different depths and non-linearities for, respectively, models in (1-2-3). This analysis aimed at determining whether a local kernel favors higher-frequency learning compared to models with full kernels (1–2) and the importance convolutional weights sharing (3).
- Repeat the analysis across multiple datasets (MNIST, FashionMNIST, SVHN, CIFAR10, and CIFAR100) to test if the phenomenon is dependent on the dataset statistics.
- Plot the fraction of energy outside a fixed frequency interval of $\left[-\frac{k}{2},+\frac{k}{2}\right]$, divided by the total energy, for each kernel (which we called κ_*high*_). This analysis aimed to assess whether smaller convolutional kernels favor concentration of energy in high frequencies for the learned network's weights. The specific value of *k* was chosen arbitrarily, as we are interested in observing the overall trend.

**Figure 3:**
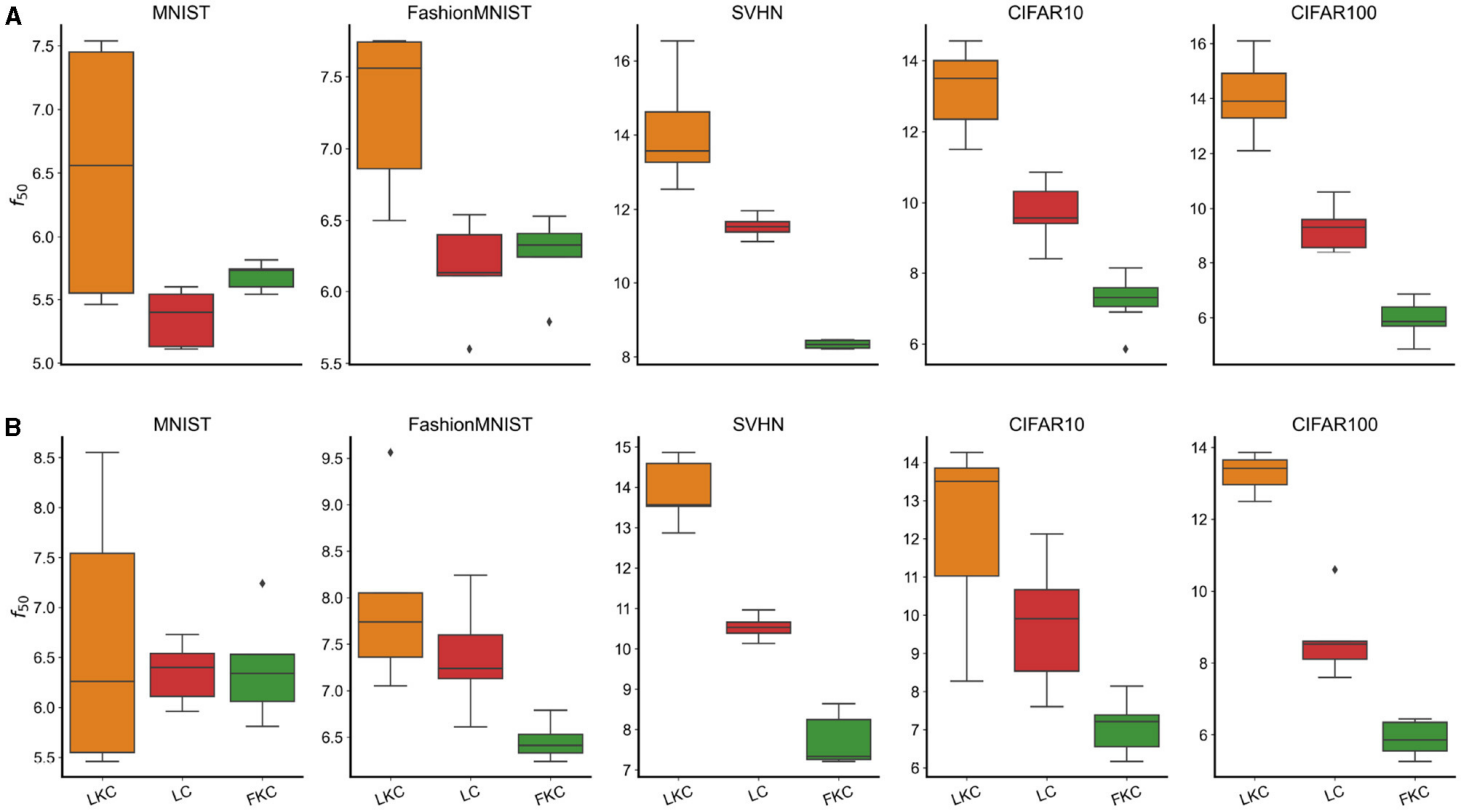
**(A)** Half Power Frequency (*f*_50_) of the Fourier transform of the weights $\hat{\beta }$ for various models and datasets. In specific: Convolutional models with Local Kernels (LKC), Locally Connected (LC), and Full Kernel Convolutional (FKC) trained on MNIST, Fashion Mnist, SVHN, CIFAR10, and CIFAR100. **(B)** Half Power Frequency (*f*_50_) of the adversarial perturbation $\hat{\delta }$ for the same models and datasets.

The results depicted in Figure 3 confirm the validity of the IFR hypothesis. Notably, we observe that models with full kernels exhibit a lower *f*_50_ compared to those with local kernels (a). This trend holds true for all models that possess a significant content of useful high-frequency features, which accounts for the distinct behavior observed in MNIST and FashionMNIST datasets (Rahaman et al., 2019). Furthermore, this phenomenon appears to be independent of the chosen dataset, as convolutional architectures with local kernels consistently demonstrate higher *f*_50_ values, albeit at different levels. Our findings also underscore the *pivotal role of convolutional weights translational symmetry in determining the frequency bias*. For instance, the *f*_50_ values of locally connected models with the same kernel size (LC) differ significantly from those of local kernel convolutional models. The same observations are applicable to adversarial perturbations (b).

**Figure 4:**
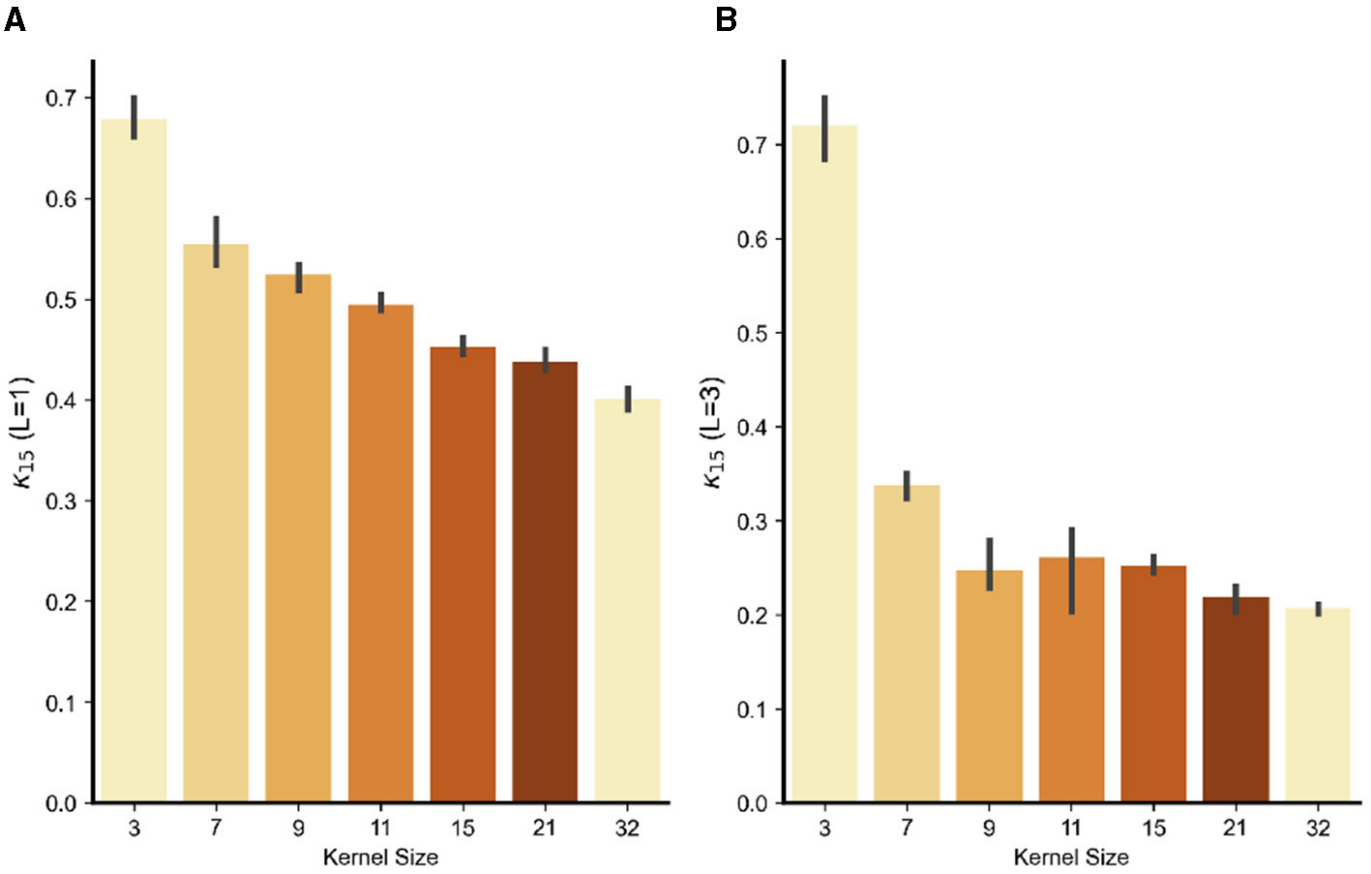
Concentration of energy κ_*high*_ for *k* = 15 (the energy cut-off to consider high energy frequencies) for the input-output weights β versus kernel size (3,7,9,11,15, 21, or 32). Results are obtained averaging five models. All models were trained for 40 epochs on Grayscale CIFAR10. **(A)** One hidden layer models and **(B)** three hidden layer models.

To further test the dependence of the frequency bias from the kernel size, Figure 4 reports the fraction of energy κ_*high*_(15) of $\hat{\beta }$ and $\hat{\delta }$ for the models with increasing kernel size. As predicted by the IFR, *the fraction is a decreasing function of the kernel size* (a) and, interestingly, as the model goes deeper, the phenomenon is exacerbated (b). This is one of the main results of the paper and clearly illustrates that the adversarial attacks high-frequency content increases with smaller kernel size. All together these results confirm the validity of the IFR.

Regarding the kernel size, from a theoretical point of view, we offer an explanation via the Fourier Uncertainty Principle—i.e., a space-limited kernel *cannot* be band-limited in frequency domain—as the origin of the frequency bias. The reasoning can be made rigorous for a linear convolutional model with local kernels by a straightforward extension of the results in Gunasekar et al. (2018). To do so, let us note first that, in the case of convolutional networks, the linear predictor β in Equation (1) can be rewritten as $\beta :={⋆}_{l=1}^{L-1}w_{l}$, where *w*_*l*_ are the kernel for layer *l* and ⋆ indicates convolution. Then we have the following: Theorem 1. Decreasing the kernel size of each convolutional filter *w*_*l*_ results in an increased concentration of energy in high frequencies for $\hat{\beta }$.

A rigorous mathematical treatment can be found in the Supplementary Section 2. The key intuition is that, due to the Fourier Uncertainty Principle, decreasing the support of the convolutional filter *w*_*l*_ at layer *l* causes an increase of its high frequency energy content.

### 3.3 Testing of the different implicit biases via the injection of hidden shortcut features

In this section we present an alternative and indirect demonstration of the validity of the IFR for the models considered in the previous section i.e., convolutional with (1) full or (2) local kernels and (3) locally connected models. Our approach draws inspiration from the field of *steganography*, which focuses on introducing shortcut features in images in a visually imperceptible manner (Cheddad et al., 2010). We introduce class-correlated shortcut features in the Fourier space of each CIFAR-10 train and test set image. Depending on the frequency range of these features (low, medium, or high), convolutional models with reduced kernel sizes are expected to demonstrate improved test accuracy, in line with the findings discussed in Section 3.2. Specifically, we performed a hidden features experiment, and localize the information in the low, medium, or high frequencies by introducing the class-dependent signals characterized by frequency in specific bands of the spectrum of the training and testing set of CIFAR10 (see Section 2 for methodological details).

Table 1 shows the performance of the linear models where class-dependent features with different frequencies (Low, Medium or High) were added to the images. We observe that all models are able to use the low-frequency shortcut features in order to perform the task achieving 100% accuracy. However, when the cheat signal is introduced in the medium and high frequencies some models perform better than others. In particular, full kernel convolutional models struggle in selecting the signal as they have an average variation in performance for both medium and high frequencies of only 1.5%. In contrast, the convolutional models with local kernels demonstrate superior performance in medium and high frequencies with an average variation in performance of ≈31%. Lastly, Locally connected kernels have an average change in performance of ≈13%.

**Table 1:** Performance on CIFAR10 dataset with class-revelant information introduced at different frequency bands.

**Models**	**Baseline**	**Low frequency**	**Medium frequency**	**High frequency**
Full kernel convolution (*L* = 1)	39.8	100.0	41.03	41.38
Full kernel convolution (*L* = 3)	40.1	100.0	39.80	40.97
Local kernel convolution (*L* = 1)	41.8	100.0	49.99	53.60
Local kernel convolution (*L* = 3)	42.5	100.0	**94.17**	**98.47**
Locally connected (*L* = 1)	40.7	100.0	44.39	46.23
Locally connected (*L* = 3)	42.2	100.0	47.36	54.73

Low Frequency (ϵ = 0.5), Medium Frequency (ϵ = 5*e*−02), and High Frequency (ϵ = 5*e*−04) (see Section 2 for details). Different ϵ's were chosen to match the signal to noise ratio of those frequencies bands and make the task as difficult as possible.

These experiments not only confirm the existence of an implicit bias of the models as characterized in the previous sections, but also demonstrate that, when useful high-frequency information is present, models with local convolutions are more adept at capturing these features compared to other parameterizations.

### 3.4 Expanding to other state-of-the-art machine learning models and Imagenet dataset

Here we further investigate the high-frequency bias exhibited by convolutional models with local kernels when training is done on one of the most complex image recognition datasets available, Imagenet (Deng et al., 2009). Additionally, we aim to examine whether other high-performance deep models without convolutions, such as Vision Transformers (ViT), exhibit less bias toward high-frequency features in their weights and attacks. ViT have performed on par with convolution-based architectures in many tasks including object recognition (Dosovitskiy et al., 2020). Interestingly, recent work has shown that ViTs can be more robust to high frequency adversarial perturbations than ResNets (Shao et al., 2021).

We selected models with different parameterizations from the timm package (Wightman, 2019). These include Convolution-Based Models, Vision Transformers, Hybrid ViT and Convolutional Models, and MLPs models (for detailed information on the specific pretrained timm models, refer to Supplementary Sections 1.1, 1.3 for details).

**Figure 5:**
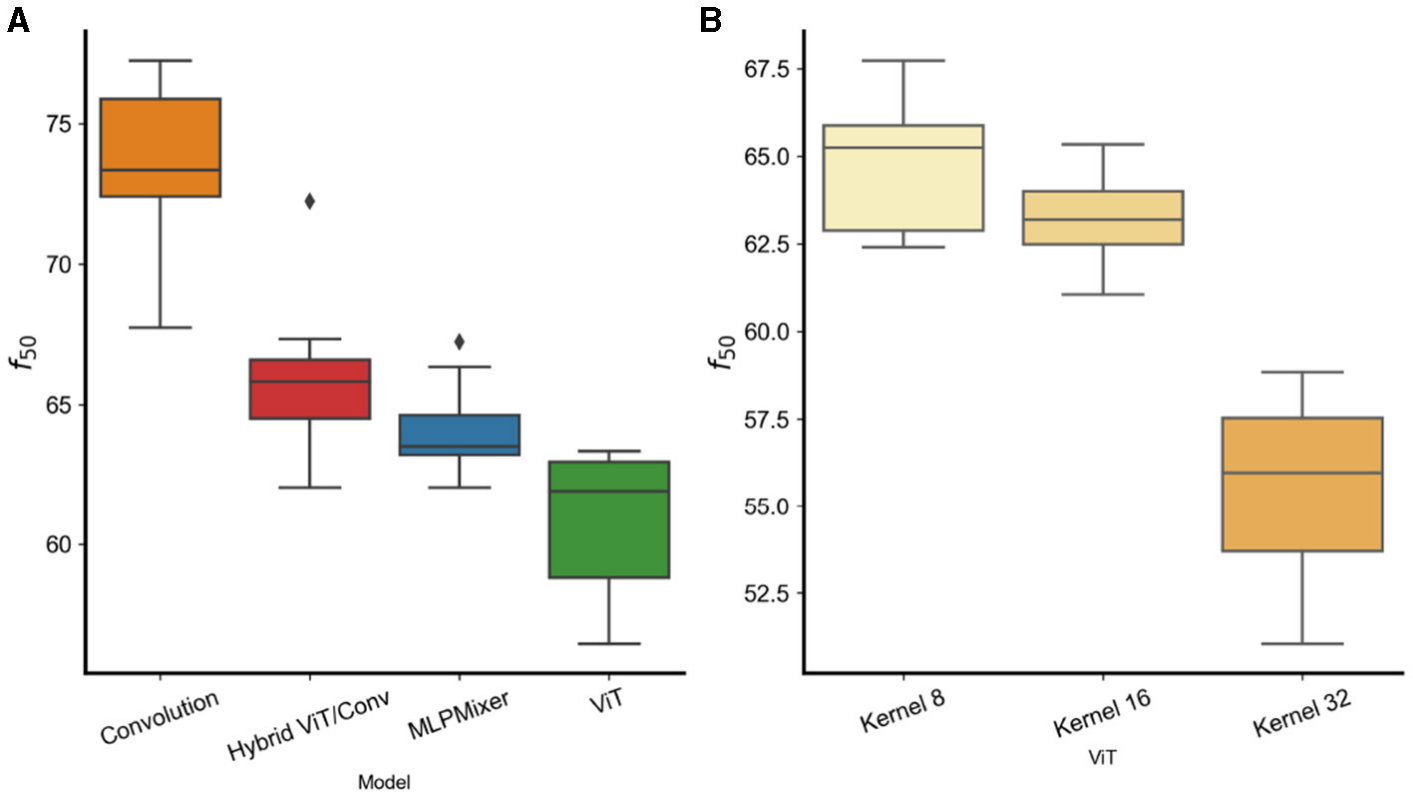
**(A)** *f*_50_ for different models trained on Imagenet. **(B)** ViT models with different kernel word/patch sizes.

Figures 5A, B shows the *f*_50_ energy of $\hat{\delta }$. We observe that pure convolutional models exhibit a stronger energy concentration in the higher frequencies, confirming the results of previous sections. Moreover in (b) shows that models with larger first layer word/patch size kernels [ViT(32)] have more energy in the low frequencies compared to models with smaller kernel sizes [ViT(16), ViT(8)]. This confirms the results in Section 3.2 with models trained on CIFAR10. Interestingly, the work in Park and Kim (2022) showed that self-attention layers can produce models with lower frequency preference and that a combination of the Vision Transformers with Convolutions generates a compromise frequency preference. Here we confirm those findings showing that hybrid models already have a *f*_50_ in between ViTs and Convolution models and also find that also MLP-Based models have similar energy distribution compared to hybrid models. All together these results show that, regardless of the dataset, convolution-based models have a preferences toward higher frequency features compared to the non-convolutional counterparts.

## 4 Discussion and conclusions

In this study, we provide both empirical and theoretical evidence to support the hypothesis that the convolutional architecture of modern high-performance networks, particularly the locality of convolutional kernels, plays a significant role in the emergence of high-frequency adversarial examples. To explore this phenomenon, we first validated theoretical findings related to the implicit bias of deep linear models with full connections, whether fully connected or convolutional, as outlined by Gunasekar et al. (2018). We then extended these results to nonlinear models and established a correlation between the end-to-end weights of the model and the adversarial perturbations.

Afterwards, our focus shifted to convolutional models with local kernels, and we have provided empirical and theoretical evidence (limited to linear models) to demonstrate their bias toward high-frequency features compared to other model parameterizations. crucially, we have highlighted the importance of convolutional translation symmetry (weight sharing) in our findings.

Through experiments with different datasets, we present evidence that translational symmetric convolution-based models exhibit higher energy in the high frequencies when there is significant useful high-frequency information present. This finding departs from the conventional understanding of adversarial perturbations and generalization, where high-frequency adversarial attacks are assumed to be solely determined by dataset statistics. Instead, our results demonstrate that even in datasets with higher frequency information, such as CIFAR10, CIFAR100, and SVHN, models with non-convolutional architectures (e.g., fully connected, locally connected, and Vision Transformers) exhibit fewer high-frequency adversarial attacks.

In order to further examine the network bias, we also conducted a novel steganography experiment. The results of this experiment provided compelling evidence that the bias observed in linear models extends to nonlinear models as well. Additionally, we found that this bias significantly influences the ease with which the models can learn specific features (Section 3.3). These findings open up new and exciting avenues for investigating the model's ability to generalize across various bases, not limited to Fourier.

Through comparison with other high-performing models, we demonstrated that Vision Transformers (ViTs) with smaller kernel sizes also exhibit higher energy in the high frequencies for both CIFAR10 and ImageNet trained models. This suggests that our findings are robust and applicable across different datasets regardless of their statistics. Moreover they offer valuable information for designing and understanding the implicit biases in various model architectures.

We firmly believe that understanding such biases can guide models toward effectively leveraging the most beneficial set of features while simultaneously reducing the vulnerability of modern neural networks to adversarial attacks. Although our study focuses on convolutional architectures we believe that shedding light on the interplay between the model architecture and adversarial robustness is essential to the development of more reliable and secure neural network systems. This research opens up new avenues for improving the overall performance and safety of deep learning models in real-world applications.

## Data availability statement

The raw data supporting the conclusions of this article will be made available by the authors, without undue reservation.

## Author contributions

JC: Writing—review & editing, Writing—original draft, Visualization, Validation, Software, Methodology, Investigation, Formal analysis, Data curation, Conceptualization. YJ: Writing—review & editing, Writing—original draft, Visualization, Validation, Investigation. RP: Writing—review & editing, Writing—original draft, Visualization, Validation, Investigation. SD: Writing—review & editing, Writing—original draft, Supervision, Methodology, Conceptualization. WB: Writing—review & editing, Writing—original draft, Supervision, Methodology, Conceptualization. FA: Writing—review & editing, Writing—original draft, Supervision, Project administration, Methodology, Formal analysis. AP: Formal analysis, Writing—review & editing, Writing—original draft, Supervision, Resources, Project administration, Funding acquisition, Conceptualization.
