# Supplementary material for: Translational symmetry in convolutions with localized kernels causes an implicit bias toward high frequency adversarial examples
Source: Front Comput Neurosci. 2024 Jun 20;18:1387077. doi: 10.3389/fncom.2024.1387077 (PMC11222393; doi:10.3389/fncom.2024.1387077)
Supplement: Supplementary file 1 [file Data_Sheet_1.pdf]

Translational Symmetry in Convolutions with Localized Kernels Causes an Implicit Bias towards High Frequency Adversarial Examples

Josue O. Caro[1,*], Yilong Ju [1,2], Ryan Pyle [1,2], Sourav Dey[3],Wieland Brendel[4], Fabio Anselmi[1,5,#],and Ankit B. Patel[1,2,#]

[1]*Department of Neuroscience, Baylor College of Medicine, Houston, 77030, USA.*

[2]*Department of Electrical and Computer Engineering, Rice University, Houston, 77005, USA*

[3]*Manifold AI*

[4]*University of Tübingen, Max Planck Institute for Intelligent Systems, Germany*

[5]*Department of Mathematics, Informatics and Geosciences, University of Trieste, Via Alfonso Valerio 12 Bld H2bis, Trieste,34127, Italy*
and Massachusetts Institute of Technology (MIT), 77 Massachusetts Ave, Cambridge,02139, MA,USA

† *co-first authors*

senior authors

Correspondence*:
Josue Ortega Caro
josue.ortegacaro@yale.edu

1 SUPPLEMENTARY MATERIAL

1.1 Model Architecture

Supplementary Table 1. Model Architectures

Model Architecture	# Hidden Layers	Nonlinearity	Channels
Fully Connected	1,3	None, ReLU	3072
Local Kernel Convolution	1,3	None, ReLU	32
Full Kernel Convolution	1,3	None, ReLU	32
Locally Connected	1,3	None, ReLU	32

Supplementary Table 2. Model Configurations for ImageNet Trained Models. All models were pulled from the timm package.

Model	Model Type	Model timm package name
ResNet50	Convolutional	resnet50d
EfficientNet	Convolutional	tf_efficientnet_b0_ns
RepVGG	Convolutional	repvgg_b3
ConvViT	Hybrid	convit_base
ViT-ResNet50	Hybrid	vit_large_r50_s32_224
Coat	Hybrid	coat_lite_small
ResMLP (36)	MLP	resmlp_36_distilled_224
gMixer	MLP	gmixer_24_224
MLPMixer Large	MLP	mixer_b16_224
ViT (8)	ViT	vit_base_patch8_224
ViT (16)	ViT	vit_base_patch16_224
ViT (32)	ViT	vit_base_patch32_224

All ImageNet models were pull from the timm package. Furthermore, all models were trained with similar data augmentations, and adversarial attack evaluation was done with default preprocessing from $model.default_{cfg}$.

1.2 Model Performance

Supplementary Table 3. Test Accuracy for all models trained on CIFAR-10, CIFAR100, MNIST, FashionMNIST, SVHN.

	Test Accuracy (%)				
Models	FashionMNIST	MNIST	SVHN	cifar10	cifar100
Fully Connected	86.9	92.0	26.5	39.6	15.8
Full Kernel Convolution	86.6	91.0	28.7	40.5	17.7
Locally Connected	86.4	92.0	28.5	40.7	18.3
Local Kernel Convolution	86.4	92.0	28.2	40.2	16.1
Deep Full Kernel Convolution	86.5	91.8	26.0	41.7	18.9
Deep Fully Connected	86.7	92.1	23.8	39.2	14.7
Deep Locally Connected	84.1	92.0	29.0	41.8	18.8
Deep Local Kernel Convolution	86.1	92.0	27.7	39.9	14.9
Fully Connected (ReLU)	88.4	97.2	77.6	43.6	15.4
Full Kernel Convolution (ReLU)	84.8	92.8	85.9	47.7	19.2
Locally Connected (ReLU)	87.8	95.7	82.4	53.8	22.5
Local Kernel Convolution (ReLU)	90.5	98.0	83.2	59.7	29.0
Deep Full Kernel Convolution (ReLU)	87.8	97.4	86.3	51.7	22.2
Deep Fully Connected (ReLU)	89.1	97.9	67.3	50.8	10.5
Deep Locally Connected (ReLU)	86.5	96.0	86.9	57.9	18.3
Deep Local Kernel Convolution (ReLU)	91.7	98.8	86.7	66.0	29.6

Supplementary Table 4. Test Accuracy for all models trained on ImageNet.

model	top1	top5
vit_base_patch8_224	85.794	97.794
vit_base_patch16_224	84.528	97.294
vit_large_r50_s32_224	84.424	97.166
coat_lite_small	82.304	95.848
convit_base	82.286	95.938
resmlp_36_distilled_224	81.154	95.488
vit_base_patch32_224	80.722	95.566
resnet50d	80.522	95.162
repvgg_b3	80.496	95.264
tf_efficientnet_b0_ns	78.658	94.378
gmixer_24_224	78.036	93.670
mixer_b16_224	76.612	92.228

1.3 Training Hyperparameters

Supplementary Table 5. Learning rates for the various models considered on CIFAR-10. All other hyper-parameters were fixed.

Model Architecture	Learning Rate	Batch Size	Learning Rate Drop
Full Kernel Convolution	.01	128	Yes
Fully Connected	.01	128	Yes
Locally Connected	.01	128	Yes
Full Kernel Convolution	.002	128	Yes
Lokal Kernel Convolution	.002	128	Yes

Supplementary Table 6. Learning rates for the various models considered on CIFAR-100. All other hyper-parameters were fixed.

Model Architecture	Learning Rate	Batch Size	Learning Rate Drop
Local Kernel Convolution	.01	128	Yes
Fully Connected	.01	128	Yes
Locally Connected	.01	128	Yes
Full Kernel Convolution	.002	128	Yes
Local Kernel Convolution	.002	128	Yes

Supplementary Table 7. Learning rates for the various models considered on MNIST. All other hyper-parameters were fixed.

Model Architecture	Learning Rate	Batch Size	Learning Rate Drop
Local Kernel Convolution	.01	100	Yes
Fully Connected	.01	100	Yes
Locally Connected	.01	100	Yes
Full Kernel Convolution	.002	100	Yes
Local Kernel Convolution	.002	100	Yes

Supplementary Table 8. Learning rates for the various models considered on FashionMNIST. All other hyper-parameters were fixed.

Model Architecture	Learning Rate	Batch Size	Learning Rate Drop
Local Kernel Convolution	.01	100	Yes
Fully Connected	.01	100	Yes
Locally Connected	.01	100	Yes
Full Kernel Convolution	.002	100	Yes
Local Kernel Convolution	.002	100	Yes

Supplementary Table 9. Learning rates for the various models considered on SVHN. All other hyper-parameters were fixed.

Model Architecture	Learning Rate	Batch Size	Learning Rate Drop
Local Kernel Convolution	.01	128	Yes
Fully Connected	.01	128	Yes
Locally Connected	.01	128	Yes
Full Kernel Convolution	.002	128	Yes
Local Kernel Convolution	.002	128	Yes

1.4 Adversarial Attack Configurations

Supplementary Table 10. Adversarial Attack hyperparameters for CIFAR10, SVHN, CIFAR100, MNIST and FashionMNIST

Attack	Metric	Learning Rate	Number of Steps	Max Norm, ϵ
Projected Gradient Descent	L_∞	0.1	1000	8.0/255.0
Projected Gradient Descent	L_2	0.1	1000	2.0
Projected Gradient Descent	L_1	0.1	200	0.1
Brendel-Bethge Attack	L_∞	1e-03	1000	-
Brendel-Bethge Attack	L_2	1e-03	1000	-

Learning Rates. All the models adversarial attacks were generated using the configuration above with the Foolbox package **?**.

2 FORMAL PROOF OF HIGH FREQUENCY BIAS

*

PROOF. Let us first concentrate on a single convolutional filter $w_l \in \mathbb{R}^D$. Given an arbitrary choice of frequency interval $\Omega := \{-k, \dots, 0, \dots, +k\}$ and space interval $S := \{-a, \dots, 0, \dots, +a\}$, we want to prove that reducing the energy fraction in the complementary set S^c implies that we must increase the energy fraction in the complementary set Ω^c. The result follows from a direct application of the Uncertainty Principle for finite-dimensional vector spaces, as shown e.g. in Ghobber-Jaming **?**. In particular let $\hat{w} \in \mathbb{R}^D$ be the coefficients of the Discrete Fourier Transform (DFT) of a convolutional filter $w \in \mathbb{R}^D$. From equation 1.2 in **?** we have

$$\|w\|_{\ell^2(S^c)} + \|\hat{w}\|_{\ell^2(\Omega^c)} \geq \|w\|_2 C(S, \Omega) \tag{1}$$

where $C(S, \Omega)$ is constant when the intervals S, Ω are fixed. Dividing both sides of the inequality by $\|w\|_2$ we have

$$\kappa(S^c) + \hat{\kappa}(\Omega^c) \geq \text{const}$$

where $\kappa(\mathcal{A}) := \|w\|_{\ell^2(\mathcal{A})}/\|w\|_2$ is the spatial energy concentration of w in the index set $\mathcal{A}$ and $\hat{\kappa}(\mathcal{B})$ is the frequency energy concentration of $\hat{w}$ in the set $\mathcal{B}$. Thus increasing the energy concentration in the spatial interval S will cause a decrease in S^c and by the inequality above an increase in $\kappa(\Omega^c)$. If we let Ω be an interval of 'low' frequencies, then we conclude there will be an increase in the energy 2concentration in the 'high' frequencies Ω^c.

The reasoning above can be extended from a single convolutional filter to the full end-to-end weights vector, $\beta := \star_{l=1}^{L-1} w_l$, as follows. Note first that, using the convolution theorem, the Discrete Fourier transform of β is the Hadamard product of the Discrete Fourier transforms of the per-layer weights w_l i.e.

$$\hat{\beta} = \hat{w}_{L-1} \odot \cdots \odot \hat{w}_1.$$

Let us consider the energy in a set of 'low' frequencies Ω:

$$\hat{\beta}_\Omega = \hat{w}_{L-1,\Omega} \odot \cdots \odot \hat{w}_{1,\Omega}.$$

Taking the ℓ_2 norm and invoking the inequality $\|a \odot b\|_2 \leq \|a\|_2 \|b\|_2$ a total of $L-1$ times we can then write

$$\kappa(\Omega, \beta) \leq \prod_{l=1}^{L-1} \kappa(\Omega, w_l).$$

Suppose now that, all else equal, we decrease the energy concentration in each spatial domain S of the per-layer filters w_l. By the reasoning above this will increase the energy concentration in frequency domain in the interval Ω^c i.e. a decrease in $\kappa(\Omega, w_l)$ for each layer l. By the last inequality this will decrease $\kappa(\Omega, \beta)$, resulting in an increase in the energy concentration in the high frequencies (Ω^c) for β.

LEMMA 1. *Concentrating the kernel energy in spatial domain increases the implicit regularization term in the optimization in [Gunasekar]:*

$$\forall a' < a : R_{BWC;a'}(\beta) \geq R_{BWC;a}(\beta)$$

PROOF. Reducing filter size K will increase energy in high freqs i.e. $\forall K' < K : \kappa_{high}(\beta; K') > \kappa_{high}(\beta; K)$. This means that the space-limiting constraints only grow more stringent as we reduce

K, implying that the result of the optimization problem for the implicit regularizer will only increase in cost i.e. $\forall K' < K : R_{BWC;K'}(\beta) \geq R_{BWC;K}(\beta)$ for any candidate linear predictor β. (Note that this does not refer to the learned features β^* which actually depends on the training data as well). In summary, all else being equal, reducing the kernel size K causes/induces a bias towards more concentration of energy in higher frequencies in β.

In summary reducing the kernel size causes/induces a bias towards more concentration of energy in higher frequencies in β.
